# Missense variants in *SLC9A6* cause partial epilepsy without neurodevelopmental delay

**DOI:** 10.1186/s13023-025-03924-9

**Published:** 2025-07-28

**Authors:** Jun-Ping Jiao, Hong-Wei Zhang, Xi-Zhong Zhou, Shu-Juan Tian, Li Gao, Bing-Mei Li, Jun-Xia Luo, Jie Wang, Song Lan, Bin Li, Wei-Ping Liao

**Affiliations:** 1 Department of Neurology, Institute of Neuroscience, Key Laboratory of Neurogenetics and Channelopathies of Guangdong Province, The Second Affiliated Hospital, Ministry of Education of China, Guangzhou Medical University, Guangzhou, China; 2 Department of Neurology, Neuromedical Technology Innovation Center of Hebei Province, Brain Aging and Cognitive Neuroscience Laboratory of Hebei Province, the First Hospital of Hebei Medical University, Shijiazhuang, Hebei China; 3 Department of Epilepsy Center, Children’s Hospital Affiliated to Shandong University, Jinan, Shandong China; 4 Department of Pediatrics, Zhujiang Hospital of Southern Medical University, Guangzhou, China; 5 Department of Pediatrics, Henan Provincial People’s Hospital, Henan, China; 6 Department of Neurology, Maoming People’s Hospital, Maoming, China

**Keywords:** *SLC9A6* gene, Christianson syndrome, Epilepsy, Whole exome sequencing, Genotype-phenotype correlation

## Abstract

**Background:**

The *SLC9A6* gene encodes a monovalent sodium-selective sodium/hydrogen exchanger that is essential in regulating endosomal PH and volume. *SLC9A6* variants are associated with Christianson Syndrome, a severe neurodevelopmental disorder that is accompanied by seizures. It is unknown whether *SLC9A6* variants are associated with milder phenotypes.

**Method:**

Trio-based whole-exome sequencing was performed in unrelated cases (families) with epilepsy without acquired causes. Previously reported *SLC9A6* variants were reviewed to analyze the mechanism underlying phenotype variations.

**Results:**

Five hemizygous variants, including three null and two missense variants, were identified in five males. All the variants were absent in the gnomAD-all populations and the missense variants were predicted to be damaging by multiple in silico tools. The three patients with null variants presented with refractory epilepsies and severe developmental delay; one patient with missense variant in the transmembrane region showed refractory epilepsies and speech delay; and one patient harboring missense variant located in the loop region achieved seizure-free with favorable outcome. Further analysis revealed that the proportions of brain atrophy, microcephaly, and movement disorders in patients with missense variants were significantly lower than that of patients with null variants, suggesting a genotype-phenotype correlation. Additionally, previously reported missense variants in the pore/transmembrane region led to Christianson Syndrome, whereas variants outside these regions were associated with milder phenotype, suggesting a sub-regional effect.

**Conclusion:**

Missense variants in *SLC9A6* are associated with mild partial epilepsies. The genotype-phenotype correlation and molecular sub-regional effect of *SLC9A6* help in explaining the mechanisms underlying phenotypic variations.

**Supplementary Information:**

The online version contains supplementary material available at 10.1186/s13023-025-03924-9.

## Introduction

The *SLC9A6* gene (OMIM* 300231), also known as NHE6 gene, is located on chromosome Xq26.3 and encodes a monovalent sodium-selective sodium/hydrogen exchanger (NHE) [1, 2]. The *SLC9A6* protein is mainly expressed in human brain tissues [1, 3]. It is found in the membranes of intracellular organelles such as mitochondria and endosomes, playing an essential role in regulating endosomal pH and volume [4–7]. Homozygous KO mice show postnatal lethality [8, 9] suggesting an essential role of *SLC9A6* in neuronal development.

Previously, *SLC9A6* variants have been reported to be associated with X-linked syndromic intellectual developmental disorder (MRXSCH, OMIM # 300243), as known as Christianson Syndrome (CS) [10–14]. CS is characterized by early-onset refractory epilepsy, severe intellectual disability (ID), and global developmental delay (GDD) [12, 13, 15, 16]. It is unknown whether *SLC9A6* variants are associated with pure epilepsies, and the mechanisms underlying phenotypical variations remain elusive [17]. 

In the present study, trio-based whole exome sequencing (WES) was performed in cases (families) of epilepsy without acquired causes. *SLC9A6* hemizygous variants, including three null and two missense variants, were identified in five males with epilepsy. We further reviewed all previously reported *SLC9A6* variants and analyzed their molecular heterogeneity, aiming to explore the mechanism underlying phenotypical variation.

## Materials and methods

### Participants

The patients in this study were recruited from a multicenter cohort via the China Epilepsy Project 1.0 (https://epg1.cn/), which included fifteen cohorts subclassified by clinical phenotypes. Detailed clinical information, including clinical phenotypes, age at onset of seizures, seizure types, duration and frequency of seizures, family history, treatment status, prognosis, routine and neurological physical examinations, as well as brain magnetic resonance imaging (MRI), was collected. Electroencephalogram (EEG) monitoring, including hyperventilation, intermittent photic stimulation, eye-opening test, and sleep recording, was analyzed by at least two qualified EEG technicians. The diagnosis of epilepsy and seizure classification followed the criteria established by the International League Against Epilepsy (ILAE) [18–22]. Patients with acquired causes of epilepsy were excluded.

### WES

The study collected blood samples from both the probands and their parents, and genomic DNA was extracted from the peripheral blood using the Qiagen Flexi Gene DNA kit (manufactured by Qiagen, Hilden, Germany) in accordance with the manufacturer’s instructions. A trio-based WES was performed on the MGI 2000 platform by BGI-Shenzhen. The WES consisted of paired-end reads with 100 bp sequencing, resulting in an average depth of coverage of 100-150X and a target region coverage exceeding 98%. The deep sequencing data were aligned to the GRCh37 build (hg19) reference genome. Following this alignment, single-nucleotide variants and insertion/deletion variants were identified and annotated using the Genome Analysis Tool Kit as previously reported [23, 24]. 

### Genetic analysis

To identify potential pathogenic variants in each individual case, a tailored strategy was employed. Initially, rare variants with a minor allele frequency below 0.005 in publicly available databases such as the 1,000 Genomes Projects, Exome Aggregation Consortium, and gnomAD were firstly prioritized. Then, potentially disease-causing variants, such as canonical splicing, nonsense, frameshift, in-frame indels, missense, and initiation codon variants, were selected for further analysis. Subsequently, stratified minor allele frequency (MAF) criteria were applied: *de novo*, hemizygous, and homozygous variants should not be found in control populations in gnomAD. For biallelic variants, the product of the frequencies of the two alleles in gnomAD should be less than 1 × 10^− 6^, which is significantly lower than the expected probability of such an occurrence in the current gnomAD population (1/141456 = 7 × 10^− 6^). Ultimately, the candidate variants were refined through the application of stringent criteria sourced from the Genetic Dependence & Pathogenicity Database (www.gdap.org.cn), encompassing four pivotal facts of the gene profiles:

1) Tissue-specific expression: a prerequisite for a gene to be considered epilepsy-causing is its expression within the brain (inclusion criterion). Additionally, alternative explanations, such as ectopic expression or remote toxic effects stemming from abnormal metabolic byproducts, were also taken into consideration.

2) Exclusion of conflicting gene-disease associations: genes that have been definitively linked to diseases that are incompatible with the epilepsy phenotype were systematically excluded from further consideration (exclusion criterion).

3) Gene intolerance to variants: the probability of a gene being intolerant to heterozygous or homozygous loss-of-function variants (pLI/pRec) was evaluated. Specifically, genes with a pLI score of 0.9 or higher, harboring dominant variants, and those with both pLI and pRec scores of 0.9 or higher, along with a pNull score of 0.1 or lower in the context of recessive variants, were given particular attention.

4) Phenotypic consequences of genetic manipulation: whether genetic knockout or knockdown experiments resulted in brain-related phenotypes was explored as a way of validating the pathogenicity of genes.

After applying the filtering criteria, genes with recurrent variants were chosen for further investigation to elucidate their specific gene-disease associations. One of the candidate genes that emerged from our analysis was *SLC9A6*. Sanger sequencing was employed to validate our findings and confirm the origin of the identified variants. All the identified variants within the *SLC9A6* gene in this study were annotated using the reference transcript NM_006359.3.

### Damaging effects of *SLC9A6* variants

We used nine commonly used in silico tools, including SIFT, FATHMM_MKL, Mutation-Taster, CADD, Geno-Canyon, fitCons, M_CAP, GERP++, and SiPhy, to predict the impacts of missense variants. The results were obtained from the VarCards database (http://genemed.tech/varcards), an integrated genetic and clinical database for coding variants in the human genome.

To predict the impact of missense variants on molecular structure, we utilized the SWISS-MODEL to conduct protein modeling. The three-dimensional protein structure and hydrogen bonding alterations of missense variants were visualized and analyzed by PyMOL 2.6. The changes of the protein stability were assessed using the free energy stability change (DDG, Kcal/mol) value. Amino acids were annotated to the reference protein NP_006350.

### Exploring of mechanism underlying phenotype variations

To reveal the underlying mechanism of phenotype variations, we first reviewed all the relevant literature about *SLC9A6* variants reported previously. All variants were obtained from PubMed [4] and the Human Gene Mutation Database [5] up until March 2023 (HGMD, http://www.hgmd.cf.ac.uk/ac/index.php). Variants, such as nonsense, frameshift, splicing variants in classical soil 1 or 2, start codon, single or multiple exon deletion variants were treated as null variants. Then, we explored (1) genotype-phenotype correlation by comparison of proportions of patients with cerebral atrophy, microcephaly, and motor disorders in patients with null variants and missense variants; and (2) sub-molecular effect, the locations and damaging effects of previously reported missense variants and their phenotype severity.

### Statistical analysis

Statistical analysis was performed using GraphPad Prism software 10.0. The data are expressed as mean ± SEM. Statistical comparisons were conducted using Student’s t-tests and Chi-square tests, with a significance level set at *p* < 0.05.

## Results

### Identification of *SLC9A6* variants

**Fig. 1:**
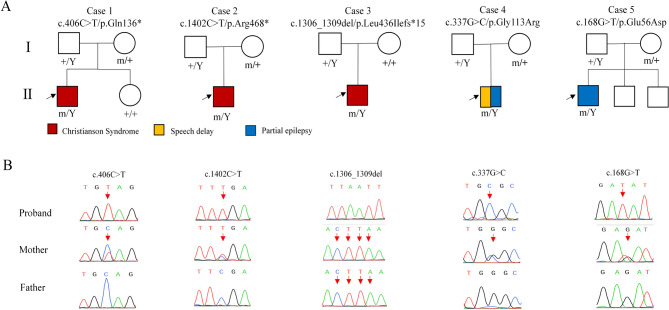
Genetic characterization of the cases with *SLC9A6* variants. (**A**) Pedigrees of the cases with *SLC9A6* variants and their corresponding phenotypes. (**B**) DNA sequence chromatogram of the *SLC9A6* variants. Arrows indicated the positions of the variants

Five hemizygous *SLC9A6* variants, including three null (c.406 C > T/p.Q136*, c.1402 C > T/p.R468*, and c.1306_1309del/ p.L436Ifs*15) and two missense variants (c.168G > T/p.E56D and c.337G > C/p.G113R), were identified in five unrelated individuals (Fig. 1; Table 1). The variant c.1306_1309del/p.L436Ifs*15 arose d*e novo* and the other variants were inherited from unaffected mothers, consistent with an X-linked recessive inheritance pattern.

**Table 1:** Clinical features of the cases with *SLC9A6* variants

Case ID	Variant (NM_006359.3)	Gender	Age	Seizure onset	Seizure type	ASMs	Seizure outcome	EEG	Cerebral atrophy	ID	Microcephaly	Motor development	Strabismus	Diagnosis
1	c.406 C > T/ p.Gln136*	Male	8 y	9 m	TS, GTCS, AS, (6–7 times/ d)	VPA, LEV, TPM LTG, CLB	2–3 times/ d~ 1/ m	ESES (5 y)	Yes	Severe	Yes	Speech delay, unable to stand	Yes	CS
2	c.1402 C > T/ p.Arg468*	Male	3 y	1 y	GTCS (1–2 times/d), SPS (1–2 times/ d)	LEV, PER	2–4 times/ m	Diffuse discharges (1 y)	Yes (1 y)	Severe	Yes	Unable to stand and talk	Yes	CS
3	c.1306_1309del/ p.Leu436Ilefs*15	Male	5 y	1 y 7 m	CPS, GTCS	Multiple ASMs	NA	NA	Yes (23 m)	Severe	Yes	Unable to stand and talk	Yes	CS
4	c.337G > C/ p.Gly113Arg	Male	8 y	5 y	CPS (5–6 times/ m)	PER, LEV	CPS 4–5 times/ y	Bilateral central, parietal, and temporal discharges independently (8y)	No (5 y)	No	No	Speech delay	No	Partial epilepsy
5	c.168G > T/ p.Glu56Asp	Male	17y	6 y	CPS (5 -9 times/ d)	LTG, VPA	Seizure free for 18 m	Left temporal discharges at 11 y and is normal at 13 y/15 y/ 17 y	No atrophy, hippocampal sclerosis (15 y)	No	No	Normal	No	Partial epilepsy

Abbreviations: AS, absence seizure; CLB, clobazam; CPS, complex partial seizure; CS, Christianson syndrome; d, day; ESES, electrical status epilepticus in slow-wave sleep; GDD, global developmental delay; GTCS, generalized tonic-clonic seizure; LEV, levetiracetam; LTG, lamotrigine; m, month; NA, not available; OFC, occipital frontal circumference; PER, perampanel; SPS, simple partial seizure; TPM, topiramate; TS, tonic seizure; VPA, valproic acid; y, year

All the three hemizygous null variants (PVS1) were not present in gnomAD-all populations (PM2) (Table 2). The c.406 C > T/p.Q136* and c.1402 C > T/p.R468* were predicted to be damaging by in silico tools (PP3). The c.1402 C > T/p.R468* was a previously reported pathogenic variant (PS1). The c.1306_1309del/ p.L436Ifs*15 was *de novo* origin (PS2). According to ACMG guidelines, the three null variants were evaluated to be “Pathogenic”.

**Table 2:** Allele frequencies and bioinformatics analysis of the *SLC9A6* variants identified in this study

Case No	cDNA change (NM_022168.4)	Protein change	Inheritance	MAF		In silico missense prediction									ACMG (scoring)
				gnomAD	gnomAD - EAS	SIFT	FATHMM _MKL	Mutation-Taster	CADD	Geno Canyon	fitCons	M_CAP	GERP++	SiPhy	
1	c.406 C > T	p.Gln136*	Maternal	-	-	-	D (0.913)	D (1)	D (37)	D (1)	-	-	C (5.07)	C (16.698)	*P* (PVS1 + PM2 + PP3)
2	c.1402 C > T	p.Arg468*	Maternal	-	-	-	D (0.913)	D (1)	D (37)	D (1)	-	-	C (5.07)	C (16.698)	*P* (PVS1 + PS1 + PM2 + PP3)
3	c.1306_1309del	p.Leu436Ilefs*15	De novo	-	-	-	-	-	-	-	-	-	-	-	*P* (PVS1 + PS2 + PM2)
4	c.337G > C	p.Gly113Arg	Maternal	-	-	D (0.001)	D (0.990)	D (1)	D (27.8)	D (1)	-	D (0.15)	C (5.07)	C (16.698)	US (PM2 + PP3)
5	c.168G > T	p.Glu56Asp	Maternal	-	-	T (0.655)	D (0.778)	D (0.955)	T (14.61)	D (1)	-	D (0.285)	C (2.91)	NC (8.964)	US (PM2 + PP3)

Abbreviations: ACMG, american college of medical genetics and genomics; C, conserved; CADD, combined annotation-dependent depletion; D, damaging; EAS, east asian; FitCons, the fitness consequences of functional annotation; GERP, genomic evolutionary rate profiling; gnomAD, the genome aggregation database; LP, likely pathogenic; MAF, minor allele freuency; NC, non-conserved; P, polymorphism; PM2, absent in population databases; PP3, multiple lines of computational evidence support a deleterious effect on the gene/gene product; PS1, reported pathogenic variants (identical amino acid changes); PS2, de novo in a patient with the disease and no family history; PVS1, null variants (nonsense variants, frameshift variants, splicing variants in classical soil 1 or 2, start codon variants, single or multiple exon deletions) when the pathogenic mechanism of a disease is loss of function (LOF); SIFT, sorts intolerant from tolerant; T, tolerable; US, uncertain significance

Number of algorithms predicted to be deleterious: total in silico algorithms, which was retrieved from the website http://varcards.biols.ac.cn/. Due to space limitations, only nine typical results were indicated in this table

The two missense variants were also not present in the gnomAD-all populations dataset. Additionally, at least five in-silico tools predicted these variants to be “damaging” (Table 2).

The five cases did not have any other pathogenic or likely pathogenic variants in genes known to be associated with seizure disorders [25]. 

### Locations and molecular effect of *SLC9A6* variants

**Fig. 2:**
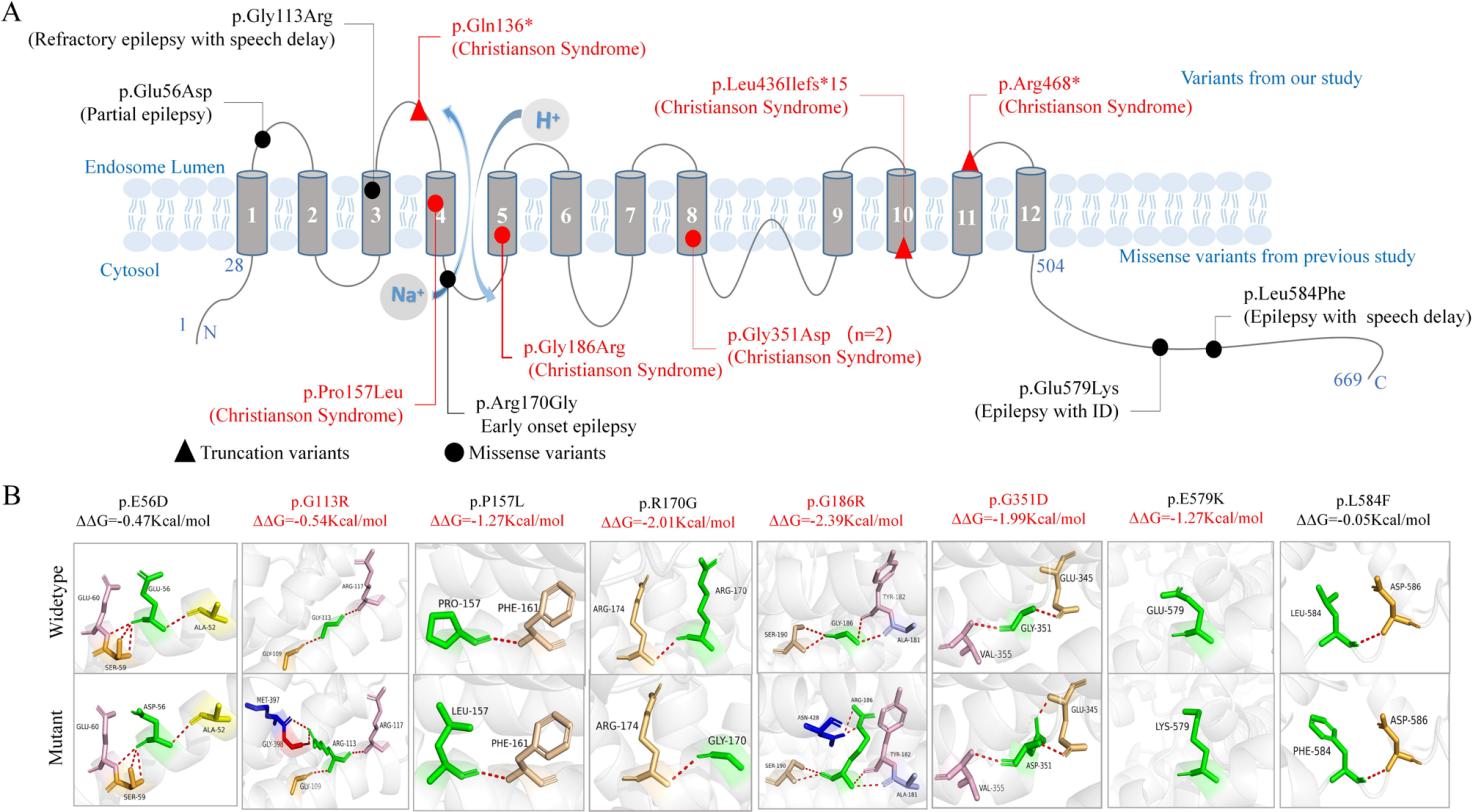
Schematic illustration of variants location, hydrogen bond changes, and protein stability prediction. (**A**) Schematic diagram of *SLC9A6* protein and the location of variants and their corresponding phenotypes. (**B**) Hydrogen bond changes and DDG values of *SLC9A6* missense variants. The red dotted line represented hydrogen bonds. Variants with hydrogen bond changes or decreased protein stability are highlighted in red

The length of amino acid of *SLC9A6* is 669. The NHE6 protein is a twelve-membrane spanning motif with the Na+/H + exchange occurring between transmembrane segments M4 and M5. There is a large and non-conservative cytoplasmic region at amino acid position 503-669 [26]. The variant c.337G > C/p.G113R, located at poor region, was associated with refractory epilepsy and speech delay, whereas the variant c.168G > T/p.E56D, which was located at loop region (Fig. 2A), was associated with pure epilepsy, suggesting a potential sub-molecular effect.

The molecular effect of the missense variants was predicted by protein modeling using PyMOL 2.6 (Fig. 2B). The variant Gly113Arg was predicted to change hydrogen bonds with nearby residues and decrease protein stability.

### Clinical features of the patients

**Fig. 3:**
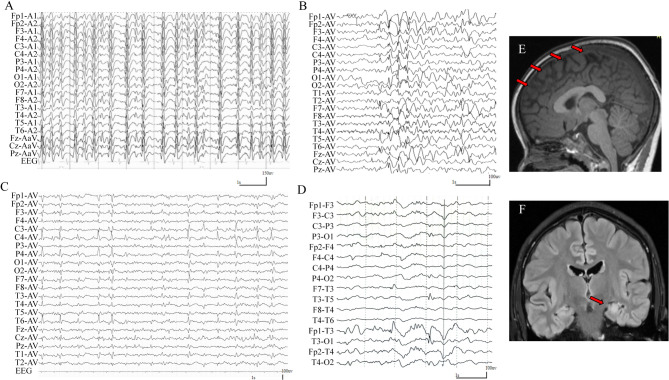
Representative interictal electroencephalography (EEG) and magnetic resonance imaging (MRI) of the cases with *SLC9A6* variants. (**A**) EEG of case 1 at the age of 5-years-old showed generalized electrical status epilepticus during slow-wave sleep. (**B**) EEG of case 2 at the age of 1-year-old showed spike-slow wave in bilateral frontotemporal and a mild degree of diffuse discharges. (**C**) EEG of case 4 at the age of 8-year-old showed sharp waves in bilateral central, parietal, and temporal discharge. (**D**) Interictal EEG of case 5 at the age of 11-year-old showed sharp waves in the left temporal region. (**E**) MRI of case 1 at the age of 3-year-old showed atrophy of the bilateral frontal and temporal poles. (**F**) MRI of case 5 at the age of 15-year-old showed hippocampal sclerosis

In the present study, five hemizygous *SLC9A6* variants were detected in five unrelated males with epilepsy and/or developmental disorders. The detailed clinical information was summarized in Table 1. The representative EEGs and MRs are shown in Fig. 3.

**Fig. 4:**
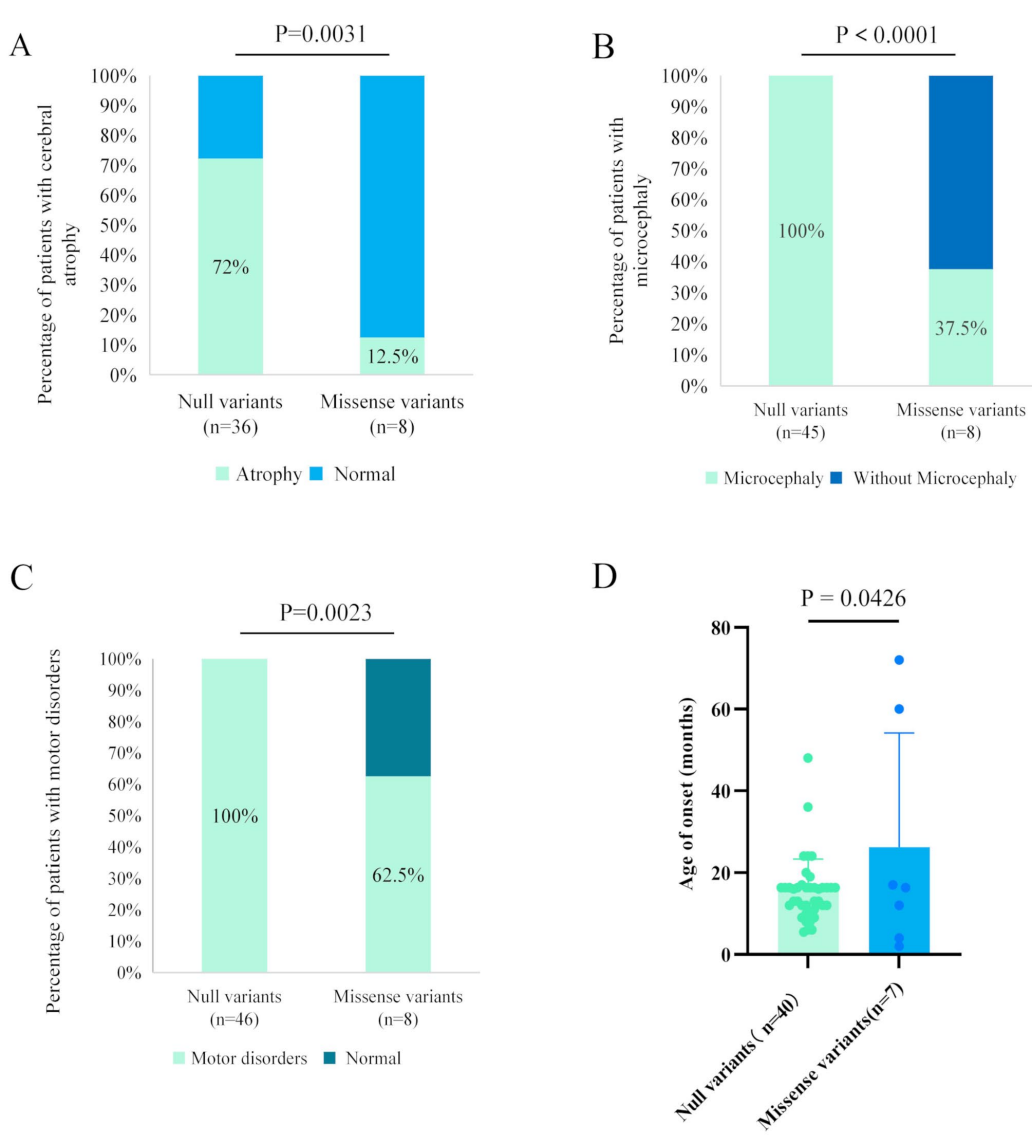
Genotype-phenotype correlation of *SLC9A6* variants. (**A**) Patients with null variants presented higher percentages of brain atrophy than those with missense variants (*p* = 0.003). (**B**) Patients with null variants presented higher percentages of microcephaly than those with missense variants (*P*<0.0001). (**C**) Patients with null variants presented higher percentages of motor disorders than those with missense variants (*P* = 0.002). (**D**) Patients with null variants presented earlier onset ages of seizures than those with missense variants (*P* = 0.0426)

The three cases with truncation variants were diagnosed as CS [27]. They presented with generalized epilepsy, with an onset age of nine months, 1 year, and 1 year and 7 months, respectively. They showed severe neurological developmental disorders and are unable to speak and walk independently. The head circumference of 44 ~ 45 cm, suggests microcephaly. The MRIs of the three cases showed cerebellar atrophy.

The two cases with missense variants presented with partial seizures, with onset ages of 5 years and 6 years, respectively. They did not present signs of microcephaly, ID, or motor developmental disorders. Case 4 showed language developmental delay. Brain MRI of case 4 was normal, and case 5 showed hippocampal sclerosis.

Two patients (cases 1 and 2) with truncation variants responded poorly to several anti-seizure medications. Among the two cases with missense variants, one achieved seizure-free (case 5) and the other one (case 4) was reduced by more than 50%.

In summary, patients with truncation variants showed CS, whereas patients with missense variants presented with epilepsy with/without developmental disorders.

### Genotype-phenotype correlation

The present study showed that *SLC9A6* missense variants are potentially associated with milder phenotypes than truncation variants. To get a full view of *SLC9A6*-associated phenotypes, we reviewed all previously reported *SLC9A6* variants and explored possible mechanisms underlying phenotype variations. Until Nov 11, 2023, including the variants identified in the present study, a total of 55 variants, including 47 null and eight missense variants, were identified in 56 cases with detailed clinical data. The detailed clinical information of previously reported patients was summarized in Table S1.

The eight missense variants were identified in nine cases. The patients presented with a variety of phenotypes, ranging from partial epilepsy with favorable outcome to severe CS. Brain atrophy, microcephaly, and motor disorders were observed in 12.5% (1/8), 37.5% (3/8), and 62.5% (5/8), respectively, in the patients with missense variants. These manifestations, which were observed in 72% (26/36), 100% (45/45), and 100% (46/46), respectively, were generally more severe and uniform in patients with null variants. The proportions of these manifestations were significantly higher in patients with null variants than that of patients with missense variants (*p* = 0.0031, <0.0001, and 0.0023, respectively) (Fig. 4A-C). In addition, the seizure onset age of patients with null variants was significantly earlier than that of patients with missense variants (*p* = 0.0426) (Fig. 4D).

### Sub-molecular effect

Given that damaging effects of variants are associated with severity of phenotype [28], we also analyzed the locations and molecular effects of previously reported *SLC9A6* missense variants. Three variants were associated with CS, among which two (p.Pro157Leu and p.Gly186Arg) were located at the pore region and one (p.Gly351Asp) was located at the transmembrane region. They were predicted to change hydrogen bonds with nearby residues and decrease protein stability. The variant p.Arg170Gly, which was associated with early-onset epilepsy, was located at the loop region and was predicted to decrease protein stability. The other two variants, p.Glu579Lys and p.Leu584Phe, which were identified in patients with epilepsy with mental retardation or speech delay, respectively, were located at the c-terminal domain, among which one (p.Glu579Lys) was predicted to decrease protein stability (Fig. 2B). These findings suggested a sub-molecular effect, i.e., variants located within the pore or transmembrane region were associated with more severe damaging, thus resulting in more severe phenotypes.

## Discussion

In the present study, we identified five *SLC9A6* hemizygous variants in five unrelated males. All the variants did not present in the gnomAD- all populations and the missense variants were predicted to be damaging by multiple in silico tools. The three patients carrying null variants presented with refractory epilepsies and severe developmental delay; one patient harboring missense variant in the transmembrane region showed refractory epilepsies and speech delay; and one patient harboring missense variant located in the loop region achieved seizure with favorable outcome, suggesting a sub-molecular correlation. The results suggested that *SLC9A6* variants are potentially associated with partial epilepsy with favorable outcomes.

The *SLC9A6* gene encodes a sodium-hydrogen exchanger that is mainly expressed in the brain [26, 3]. Variants in *SLC9A6* are associated with CS, a syndromic form of X-linked cognitive disability characterized by epilepsy, moderate to severe ID, progressive microcephaly, motor delay, and very limited language development [29–33]. Females with *SLC9A6* variants may be less severely affected [1, 10, 34–36]. Previously identified variants were mainly null variants that resulted in protein termination. Including the variants identified in this study, a total of 47 null and eight missense variants are reported so far. Further analysis suggested that the proportions of brain atrophy, microcephaly, and movement disorders in patients with missense variants are significantly lower than that of patients with null variants. Patients with missense variants presented with significantly lower seizure onset age, tended to have milder intellectual impairments, and showed a better response to medications [37, 38] suggesting a genotype-phenotype correlation.

It was noted that the seven cases with missense variants exhibited a variety of phenotypes. The *SLC9A6* gene encoded NHE6, which has 12 transmembrane helices and a regulatory C-terminal domain. There is a sodium-hydrogen exchanger (pore) region between transmembrane four and five [12]. Variants in the pore region or the transmembrane region were associated with CS, variants in the C-terminal resulted in epilepsy with mental retardation or speech delay, whereas variants in the loop region was associated with pure epilepsy, suggesting a sub-regional effect. Variants in the pore region or the transmembrane region were predicted to alter hydrogen bond with nearby residues and decreased protein stability, thus resulting in more severe phenotype.

The pLi and LOEUF of *SCL9A6* is 0.998 and 0.206, respectively, suggesting that *SLC9A6* is highly intolerant to loss of function variants. In animals, homozygous knock-out mice showed postnatal lethality. Previously reported variants are mainly null variants that result in severe neurodevelopmental delay [39–41]. The present study showed that *SLC9A6* missense variants are potentially associated with mild epilepsy without developmental delay. Therefore, the phenotype spectrum of *SLC9A6* potentially ranges from mild epilepsy with favorable outcome, to refractory epilepsy with neurodevelopmental disorders, even to early death, which is less often clinically diagnosed.

The *SLC9A6* encodes a sodium-hydrogen exchanger that is a member of the solute carrier (SLC) family, a group of membrane transport proteins comprising over 300 members. In the brain, SLC transporters are involved in the transport of various solutes across membranes, suggesting that dysfunction of SLC transporters may contribute to epilepsy and neurodevelopmental disorders. Genes like *SLC1A2*, *SLC2A1*, *SLC6A1*, *SLC12A5*, *SLC13A5*, *SLC25A22*,* SLC35A2*, and *SLC38A3* were previously found to be causative of developmental and epileptic encephalopathy [42–44]. SLC genes are also potentially associated with mild epilepsy. A typical example was *SLC2A1*, variants of which could cause late-onset epilepsy [45–48]. To our knowledge, this is the first report linking *SLC9A6* variants to mild epilepsy with favorable outcome. Under natural selection pressure, genetic variants with less damaging effects would be more common than variants with more severe damaging effects, subsequently leading to milder and common diseases. Therefore, clinically, more attention should be paid to patients with mild epilepsies.

There are several limitations to this study. First, cases with missense variants are limited and more studies are needed to clarify the whole phenotype of patients with missense variants. Second, functional investigations of the detected variants are not performed.

## Conclusion

Missense variants in *SLC9A6* are potentially associated with partial epilepsy with favorable outcomes. The genotype-phenotype correlation and sub-molecular effects may be the underlying mechanisms of phenotypic variation.

## Electronic supplementary material

Below is the link to the electronic supplementary material.

Supplementary Material 1

## Data Availability

The data that support the findings of this study are available on request from the corresponding author.

## Declarations

have been provided in this manuscript.
