# Supplementary material for: Missense variants in SLC9A6 cause partial epilepsy without neurodevelopmental delay
Source: Orphanet J Rare Dis. 2025 Jul 28;20:380. doi: 10.1186/s13023-025-03924-9 (PMC12305939; doi:10.1186/s13023-025-03924-9)
Supplement: Supplementary file 1 — Supplementary Material 1 [file 13023_2025_3924_MOESM1_ESM.docx]

**Table S1.** **Genetic and clinical features of *SLC9A6* variants previously reported**

| Variants | | Sex | Seizure type | | Age at onset | EEG Findings | Cerebral atrophy | ASMs | ID | Microcephaly | Motor development | Diagnosis | Ref |  |
| --- | --- | --- | --- | --- | --- | --- | --- | --- | --- | --- | --- | --- | --- | --- |
| Missense variants | c.470C>T/  p.Pro157Leu | M | GTCS, IS | | 4 m | Generalized discharge | No | VPA, ACTH, TPM; Effective | Yes | Yes | Hypotonia | CS | Chen et al. 2022 |  |
|  | c.508A>G/  p.Arg170Gly | M | NA | | 2 m | NA | No | NA | NA | NA | NA | Epilepsy | Ouyang X，  et al, 2021 |  |
|  | c.556G>A/  p.Gly186Arg | M | NA | | NA | NA | NA | NA | Yes | Yes | Ataxia | CS | Ilie et al, 2019 |  |
|  | c.1076G>A/  p.Gly351Asp | M | Partial seizure | | 17 m | Frontal discharges | No | VPA, LTG, OXC, Effective | Yes | No | Delayed motor development at 17m | CS | Mathieu ML,  et al, 2018 |  |
|  | c.1076G>A/  p.Gly351Asp | M | GTCS, MC,  asymmetric tonic | | 4 m-3 y | Generalized discharges | No | CBZ, CNZ, VPA, LEV, PB | Yes | Yes | No problem about hypotonia | CS | Pescosolido MF, et al, 2014 |  |
|  | c.1735G>A/  p.Glu579Lys | M | SPS, sGTCS | | 12 m | Bilateral frontotemporal interictal discharges | Yes | VPA, LEV, CLB, PIN | Yes | No | Hypotonia (3 y) | Epilepsy, ID | Padmanabha H, et al, 2017 |  |
|  | c.1752G>T/  p.Leu584Phe | M | Generalized tonic | | NA | Multifocal spikes  Slow background | No | NA | GDD | No | Hypotonia | Epilepsy,  GDD | Mir A, et al, 2022 |  |
| Null variants | c.190G>T/  p.Glu64* | M | DS, AAS, GTCS | | 4 m-3 y | "Epileptic activity" (parent report) | NA | CBZ，VPA | Regression | Yes | Hypotonia | CS | Pescosolido MF, et al, 2014 |  |
|  | c.520C>T/  p.Gln174* | M | | AA | ＜14 m | ESES | Yes | VPA, LEV, PB, TPM, LTG, MPPT; Effective | Yes | Yes | GDD | CS | Li et al, 2022 |  |
|  | c.820C>T/  p.Arg274* | M | | GTCS | 2 y | Generalized discharges | Yes | Partially responded to ASMs | Severe | Yes | Loss of walking ability (after 13 y) | CS | MigNot C, et al, 2013 |  |
|  | c.1014G>A/  P.Trp338* | M | | NA | 6 m | NA | NA | NA | NA | NA | NA | Epilepsy, early-onset | Zhao X, et al, 2022 |  |
|  | c.1710G>A/  p.Trp570* | M | | AA, GTCS, SE | 1 y | Diffuse generalized discharges, ESES | No | CBZ, LEV, VPA.  Drug resistance | Yes | Yes | Hypotonia,  a wide-based gait. | CS | Coorg R, et al, 2015 |  |
|  | c.1219C>T/  p. Gln 407* | M | | F→GTCS,  AAS | 6 m | Generalized discharges | Yes | KD, VNS, OXC, CNZ, Ineffective | Regression | Yes | Lost the abilities to crawl, pull up, and cruise at 2 y | CS | Schroer RJ,  et al, 2010 |  |
|  | c.1402C>T/  p.Arg468* | M | | GTCS, TS, CPS | 16 m | Generalized discharges | Yes | NA | Yes | Yes | Ataxia | CS | Gilfillan GD,  et al, 2008 |  |
|  | c.1402C>T/  p. Arg 468* | M | | GTCS, SE，AAS, atonic, CPS | 6-24 m | Generalized discharges | Yes | Ineffective | Yes | Yes | Five patients can’t walk, one people died. | CS, LGS | Schroer RJ,  et al, 2010 |  |
|  | c.1402C>T/  p.Arg468* | M | DS, GTCS, other | | 4 m-3 y | Normal | Yes | CBZ, CNZ, LTG, LEV | Yes | Yes | Ataxia, contractures, non-verbal | CS | Pescosolido MF, et al, 2014 | |
|  | c.1402C>T/  p.Arg468* | M | GTCS | | 4 m-3 y | Generalized discharges | NA | CLB, LTG, LEV, PB | Yes | Yes | Ataxia, hypotonia, non-verbal | CS | Pescosolido MF, et al, 2014 | |
|  | c.1402C>T/  p.Arg468* | M | GTCS | | 9 m | Generalized discharges | Yes | Drug resistance | Regressed | Yes | Unable to walk | CS | Ikeda A, et al, 2019 | |
|  | c.1473G >A/  p.Trp491* | M | GTCS | | 20 m | Generalized discharges, ESES | No | Drug resistance | Moderate to severe | Yes | Walking was acquired at 32 m | CS | Mathieu, et al. 2018 |  |
|  | c.1473G >A/  p.Trp491* | M | TS | | 4 m-3 y | Generalized fast spikes | No | VNS, CLB, DP, VPA, LEV, TPM, RUF | Regressed | Yes | Ataxia, Hypotonia, Contractures, non-verbal | CS | Pescosolido MF, et al, 2014 |  |
|  | c.1543G>T/  p.Glu515* | M | IS, TS, TCS, MC, drop seizures | | 4 m-3 y | Generalized discharges | Yes | Drug resistance | Yes | Yes | Hypotonia | CS  LGS | Pescosolido MF, et al, 2014 |  |
|  | c.1543G>T/  p.Glu515* | M | Seizure | | NA | NA | NA | NA | Yes | Yes | Ataxia | CS | Schuurs-Hoeijmakers JH, et al, 2013 |  |
|  | c.1604G>A/  p.Trp538* | M | GTCS | | 4 m-3 y | Generalized discharges | Yes | CBZ, PB, VPA | Regressed | Yes | Ataxia, Hypotonia, Hyperkinesia | CS | Pescosolido MF, et al, 2014 |  |
|  | c.1604G>A/  p.Trp538* | M | GTCS | | 4 m-3 y | Normal (1 y) | No | CLB, FBM | Regressed, | Yes | Ataxia, Hypotonia, Hyperkinesia | CS | Pescosolido MF, et al, 2014 |  |
|  | Skipping of exon 3 c.430-9_430-5delTTTTA | M | GTCS | | 3 y | Normal | Yes | VPA, Effective | Yes | Yes | Severe language delay, Hyperkinesia | CS | Masurel-Paulet A, et al, 2016 |  |
|  | c.441delG/  p.Ser147fs* | M | Multiple types of seizures | | 4 y | Generalized discharges | No | ACTH、AEDs  Ineffective | Regressed | Yes | Unable to walk and talk | CS | Takahashi et al. 2011 |  |
|  | c.477_481delCATAT/  p.Ile160Leufs*5 | M | Generalized tonic, atonic seizures | | 17 m | Generalized slow spike-wave complexes；ESES | Yes | Drug resistance | Severe | Yes | Could sit at 14m, ataxia, hypotonia, hyperkinesis | CS  LGS | Ikeda A, et al, 2019 |  |
|  | c.512_513delAT/  p.His171Leufs*60 | M | GTCS | | 1 y | Diffuse discharges | Yes | CBZ, PB | Regressed | Yes | His ability to walk was lost at 10y | CS | Gilfillan GD,  et al, 2008 |  |
|  | c.512del/  p.H171LLeufs*10 | M | TCS | | 12 m | Generalized discharges | NA | NA | Severe | Yes | DD, dystonic movements | DD,  seizures,  movement  disorder | Trump et al. 2016 |  |
|  | c.742_743delCTinsG/  p.Leu248Alafs*17 | M | AS，F, TCS,MC, | | 13 m | ESES | NA | Seizures reduced by more than 50% | Severe | Yes | Developmental regression | CS | Fung CW, et al, 2017 |  |
|  | c.1126_1130del/  p.His408Asnfs*2 | M | GTCS,  AAS | | 8 m | Generalized discharges | NA | NA | Yes | Yes | Profound DD, dystonic movements | EIEE | Trump et al. 2016 |  |
|  | c.1595_1613del/  p.Ala532Glyfs*9 | M | NA | | NA | Generalized discharges | Yes | Drug resistance | Yes | Yes | Hypotonia, GDD, non-verbal | CS | Mir A, et al, 2022 |  |
|  | exons 10-16 deletion IVS c.1141-461_UTR del | M | GTCS | | 4 m-3 y | NA | NA | VPA | Yes | Yes | Ataxia, Hypotonia, Hyperkinesia | CS | Pescosolido MF, et al, 2014 |  |
|  | c.444-451 dupAGAAGTAT/  p.Phe151fs*1 | M | DS, GTCS | | 4 m-3 y | Generalized discharges | Yes | LEV, OXC, PB, ZNS | Regressed | Yes | Ataxia, Hypotonia, Hyperkinesia | CS | Pescosolido MF, et al, 2014 |  |
|  | c.1308dupA/  p.Arg440fs*4 | M | DS, GTCS | | 4 m-3 y | Generalized discharges | Yes | LTG, LEV | Yes | Yes | Ataxia, hypotonia, non-verbal, hyperkinesia | CS | Pescosolido MF, et al,2014 |  |
|  | c.1409_1413dupCTGCC/  p.Thr472Leufs*8 | M | Suspicion | | NA | Normal | No | NA | Yes | Yes | Could not sit and stand independently; non-verbal, | CS | Yalcintepe et al. 2021 |  |
|  | c.1464dupT/  p.Thr489Tyrfs*23 | M | NA | | 10 m | NA | NA | NA | Severe | Yes | Non-verbal, can’t walk | CS | Riess A, et al, 2013 |  |
|  | c.1550dupT/  p.Leu517Phefs*5 | M | TCS | | 13 m | Discharged in frontal and temporal | No | NA | Severe | Yes | Sit at 14m, GDS: delays in large and fine motor movement | CS | Lan Y, et al, 2021 |  |
|  | c.430-1G>A | M | GTCS/sGTCS, F, drop attacks | | 4 m-3 y | Generalized discharges. | Yes | CLB, VPA, LTG, LEV, OXC, RUF | Regressed | Yes | Ataxia, Hypotonia, non-verbal, Hyperkinesia | CS | Pescosolido MF, et al,2014 |  |
|  | c.526+1G>A | M | F, GTCS, SE | | 2 y | NA | Yes | Drug resistance | Yes | Yes | Ataxia, Hypotonia, | CS | Bosemani et al, 2014 |  |
|  | c.526+1G>A | M | GTCS | | 2 y | NA | Yes | Drug resistance | Yes | Yes | Ataxia, hypotonia, | CS | Bosemani et al, 2014 |  |
|  | c.584+1G>T | M | NA | | 16 m | NA | NA | NA | Severe | Yes | Severely retarded | CS | Riess A, et al, 2013 |  |
|  | c.584+5G>A | M | FS, GTCS | | 8 m | Generalized discharges | No | NA | Yes | NA | GDD, ataxia | CS | Mercimek-Mahmutoglu S, et al, 2015 |  |
|  | c.698-2A>G | M | F, GTCS, MC, other | | 13 m | NA | NA | >50% reduction | Severe | Yes | Developmental slowdown | EE | Fung C W,  et al, 2017 |  |
|  | c.803 +1G>A | M | GTCS | | 11 m | Generalized discharges | Yes | Refractory | Severe | Yes | Walking with help at 4.5y | CS | Petraitytė et al, 2022 |  |
|  | c.1052-2A>C | M | NA | | NA | NA | NA | NA | Yes | Yes | Athetosis, generalized dystonia | DD | [Fitzgerald](https://pubmed.ncbi.nlm.nih.gov/?term=Fitzgerald+TW) T W, et al, 2015 |  |
|  | c.1141-8C>A | M | TCS, F, MC | | 10 m | Focal epileptic discharges with generalization | No | Poorly responsive | Severe | Yes | Can’t stand unaided | CS | Ieda D, et al, 2019 |  |
|  | c.1141-2A>G | M | FS, GTCS | | 11 m | ESES | Yes | VPA and LEV controlled | Severe | Yes | Can’t stand at 17m | CS | Liu X, et al, 2022 |  |
|  | c.1255-1G>A | M | TCS | | ＜2 y | Diffuse discharges, ESES | Yes | Resistance to AEDs | Severe | Yes | Ataxia and inability to walk at 7y | CS | Zanni G, et al, 2014 |  |
|  | c.1366+1G>C | M | GTCS | | 13 m | Multifocal spike and wave complexes and sharp waves | Yes | VPA+LEV  seizure-free for 1year | Regressed | Yes | Unable to talk, sit, crawl, or stand-up unaided | CS | Dong Y, et al, 2023 |  |
|  | c.1463-1G>A | M | GTCS, MC | | 12 m | Diffuse discharges | Yes | TPM, VPA, LEV, rTMS, LTG, ZNS, OXC | Yes | Yes | Ataxia, hyperkinesia | CS | Zhang et al. 2022 |  |

Aberration: AAS, atypical absence seizure; CBZ, carbamazepine; CLB, clobazam; CNZ, clonazepam; CPS, complex partial seizure; CS, Christianson syndrome; DD, Developmental disorders; DP, diazepam; DS, Dyscognitive seizures; EE, Epileptic encephalopathy; EIEE, early infantile epileptic encephalopathy; ESES, electrical status epilepticus in slow-wave sleep; F, focal seizure with or without generalization; FBM, felbamate; FS, febrile seizure; GDD, global developmental delay; GDS, gesell development schedules; GTCS, generalized tonic-clonic seizure; IS, Infantile spasms; KD, Ketogenic diet; LEV, levetiracetam; LGS, LenNox-Gastaut syndrome; LTG, lamotrigine; M, male; MC, myoclonic seizures; m, month; NA, Not available; OXC, oxcarbazepine; PB, phenobarbital; PIN, pyridoxine; RUF, rufinamide; sGTCS, secondary generalized tonic-clonic seizure; SPS, simple partial seizure; TCS, tonic-clonic seizure; TS, Tonic seizures; VPA, valproic acid; y, year; ZNS, zonisamide.

**References**

1. Hui C, Yanhui C. A case report of Christianson syndrome and discussion on its pathogenesis. Chin Clin Case Results Database. (2022) 1:E01344–4.
2. Ouyang, X., Zhang, Y., Zhang, L., Luo, J., Zhang, T., Hu, H., Liu, L., Zhong, L., Zeng, S., Xu, P., Bai, Z., Wong, L. J., Wang, J., Wang, C., Wang, B., & Zhang, V. W. (2021). Clinical Utility of Rapid Exome Sequencing Combined With Mitochondrial DNA Sequencing in Critically Ill Pediatric Patients With Suspected Genetic Disorders. Frontiers in genetics, 12, 725259.
3. Ilie, A., Gao, A. Y. L., Boucher, A., Park, J., Berghuis, A. M., Hoffer, M. J. V., Hilhorst-Hofstee, Y., McKinney, R. A., & Orlowski, J. (2019). A potential gain-of-function variant of SLC9A6 leads to endosomal alkalinization and neuronal atrophy associated with Christianson Syndrome. Neurobiology of disease, 121, 187–204.
4. Mathieu, M. L., de Bellescize, J., Till, M., Flurin, V., Labalme, A., Chatron, N., Sanlaville, D., Chemaly, N., des Portes, V., Ostrowsky, K., Arzimanoglou, A., & Lesca, G. (2018). Electrical status epilepticus in sleep, a constitutive feature of Christianson syndrome? European journal of paediatric neurology: EJPN: official journal of the European Paediatric Neurology Society, 22(6), 1124–1132.
5. Pescosolido, M. F., Stein, D. M., Schmidt, M., El Achkar, C. M., Sabbagh, M., Rogg, J. M., Tantravahi, U., McLean, R. L., Liu, J. S., Poduri, A., & Morrow, E. M. (2014). Genetic and phenotypic diversity of NHE6 mutations in Christianson syndrome. Annals of neurology, 76(4), 581–593.
6. Padmanabha, H., Saini, A. G., Sahu, J. K., & Singhi, P. (2017). Syndrome of X linked intellectual disability, epilepsy, progressive brain atrophy and large head associated with SLC9A6 mutation. BMJ case reports, 2017, bcr2017222050.
7. Mir, A., Almudhry, M., Alghamdi, F., Albaradie, R., Ibrahim, M., Aldurayhim, F., Alhedaithy, A., Alamr, M., Bawazir, M., Mohammad, S., Abdelhay, S., Bashir, S., & Housawi, Y. (2022). SLC gene mutations and pediatric neurological disorders: diverse clinical phenotypes in a Saudi Arabian population. Human genetics, 141(1), 81–99.
8. Xian L, Tingting M, Dongxiao L, Yaodong Z, Shuying L. Clinical characteristics and literature review of a case of Christianson syndrome caused by SLC9A6 gene variation. Chin J Neurol. (2022) 8:834–41.
9. Mignot, C., Héron, D., Bursztyn, J., Momtchilova, M., Mayer, M., Whalen, S., Legall, A., Billette de Villemeur, T., & Burglen, L. (2013). Novel mutation in SLC9A6 gene in a patient with Christianson syndrome and retinitis pigmentosum. Brain & development, 35(2), 172–176.
10. Zhao, X., Ning, H., Wang, Y., Zhao, G., Mei, S., Liu, N., Wang, C., Cai, A., Wei, E., & Kong, X. (2022). Genetic analysis and identification of novel variations in Chinese patients with pediatric epilepsy by whole-exome sequencing. Neurological sciences: official journal of the Italian Neurological Society and of the Italian Society of Clinical Neurophysiology, 43(7), 4439–4451.
11. Coorg, R., & Weisenberg, J. L. (2015). Successful Treatment of Electrographic Status Epilepticus of Sleep With Felbamate in a Patient With SLC9A6 Mutation. Pediatric neurology, 53(6), 527–531.
12. Schroer, R. J., Holden, K. R., Tarpey, P. S., Matheus, M. G., Griesemer, D. A., Friez, M. J., Fan, J. Z., Simensen, R. J., Strømme, P., Stevenson, R. E., Stratton, M. R., & Schwartz, C. E. (2010). Natural history of Christianson syndrome. American journal of medical genetics. Part A, 152A (11), 2775–2783.
13. Ikeda, A., Yamamoto, A., Ichikawa, K., Tsuyusaki, Y., Tsuji, M., Iai, M., Enomoto, Y., Murakami, H., Kurosawa, K., Miyatake, S., Matsumoto, N., & Goto, T. (2019). Epilepsy in Christianson syndrome: Two cases of Lennox-Gastaut syndrome and a review of literature. Epilepsy & behavior reports, 13, 100349.
14. Schuurs-Hoeijmakers, J. H., Vulto-van Silfhout, A. T., Vissers, L. E., van de Vondervoort, I. I., van Bon, B. W., de Ligt, J., Gilissen, C., Hehir-Kwa, J. Y., Neveling, K., del Rosario, M., Hira, G., Reitano, S., Vitello, A., Failla, P., Greco, D., Fichera, M., Galesi, O., Kleefstra, T., Greally, M. T., Ockeloen, C. W., … de Brouwer, A. P. (2013). Identification of pathogenic gene variants in small families with intellectually disabled siblings by exome sequencing. Journal of medical genetics, 50(12), 802–811.
15. Masurel-Paulet, A., Piton, A., Chancenotte, S., Redin, C., Thauvin-Robinet, C., Henrenger, Y., Minot, D., Creppy, A., Ruffier-Bourdet, M., Thevenon, J., Kuentz, P., Lehalle, D., Curie, A., Blanchard, G., Ghosn, E., Bonnet, M., Archimbaud-Devilliers, M., Huet, F., Perret, O., Philip, N., … Faivre, L. (2016). A new family with an SLC9A6 mutation expanding the phenotypic spectrum of Christianson syndrome. American journal of medical genetics. Part A, 170(8), 2103–2110.
16. Takahashi, Y., Hosoki, K., Matsushita, M., Funatsuka, M., Saito, K., Kanazawa, H., Goto, Y., & Saitoh, S. (2011). A loss-of-function mutation in the SLC9A6 gene causes X-linked mental retardation resembling Angelman syndrome. American journal of medical genetics. Part B, Neuropsychiatric genetics: the official publication of the International Society of Psychiatric Genetics, 156B (7), 799–807.
17. Gilfillan, G. D., Selmer, K. K., Roxrud, I., Smith, R., Kyllerman, M., Eiklid, K., Kroken, M., Mattingsdal, M., Egeland, T., Stenmark, H., Sjøholm, H., Server, A., Samuelsson, L., Christianson, A., Tarpey, P., Whibley, A., Stratton, M. R., Futreal, P. A., Teague, J., Edkins, S., … Strømme, P. (2008). SLC9A6 mutations cause X-linked mental retardation, microcephaly, epilepsy, and ataxia, a phenotype mimicking Angelman syndrome. American journal of human genetics, 82(4), 1003–1010.
18. Trump N, McTague A, Brittain H, Papandreou A, Meyer E, Ngoh A, et al. Improving diagnosis and broadening the phenotypes in early‐onset seizure and severe developmental delay disorders through gene panel analysis. J Med Genet. 2016; 53:310–17.
19. Fung, C. W., Kwong, A. K., & Wong, V. C. (2017). Gene panel analysis for nonsyndromic cryptogenic neonatal/infantile epileptic encephalopathy. Epilepsia open, 2(2), 236–243.
20. Yalcintepe, S., & Gurkan, H. (2021). Novel c.1505_1509dupCTGCC pathogenic variation in a male case with Christianson syndrome. Clinical dysmorphology, 30(1), 36–38.
21. Riess, A., Rossier, E., Krüger, R., Dufke, A., Beck-Woedl, S., Horber, V., Alber, M., Gläser, D., Riess, O., & Tzschach, A. (2013). Novel SLC9A6 mutations in two families with Christianson syndrome. Clinical genetics, 83(6), 596–597.
22. Lan, Y., Yi, S., Li, M., Wang, J., Yang, Q., Yi, S., Chen, F., Huang, L., Ruan, Y., Shen, Y., Luo, J., & Qin, Z. (2021). Case Report: Christianson Syndrome Caused by SLC9A6 Mutation: From Case to Genotype-Phenotype Analysis. Frontiers in genetics, 12, 783841.
23. Bosemani, T., Zanni, G., Hartman, A. L., Cohen, R., Huisman, T. A., Bertini, E., & Poretti, A. (2014). Christianson syndrome: spectrum of neuroimaging findings. Neuropediatrics, 45(4), 247–251.
24. Mercimek-Mahmutoglu, S., Patel, J., Cordeiro, D., Hewson, S., Callen, D., Donner, E. J., Hahn, C. D., Kannu, P., Kobayashi, J., Minassian, B. A., Moharir, M., Siriwardena, K., Weiss, S. K., Weksberg, R., & Snead, O. C., 3rd (2015). Diagnostic yield of genetic testing in epileptic encephalopathy in childhood. Epilepsia, 56(5), 707–716.
25. Fung, C. W., Kwong, A. K., & Wong, V. C. (2017). Gene panel analysis for nonsyndromic cryptogenic neonatal/infantile epileptic encephalopathy. Epilepsia open, 2(2), 236–243.
26. Petraitytė, G., Mikštienė, V., Siavrienė, E., Cimbalistienė, L., Maldžienė, Ž., Rančelis, T., Vaitėnienė, E. M., Ambrozaitytė, L., Dapkūnas, J., Dzindzalieta, R., Pranckevičienė, E., Kučinskas, V., Utkus, A., & Preikšaitienė, E. (2022). Donor Splice Site Variant in SLC9A6 Causes Christianson Syndrome in a Lithuanian Family: A Case Report. Medicina (Kaunas, Lithuania), 58(3), 351.
27. Deciphering Developmental Disorders Study (2015). Large-scale discovery of novel genetic causes of developmental disorders. Nature, 519(7542), 223–228.
28. Ieda, D., Hori, I., Nakamura, Y., Ohashi, K., Negishi, Y., Hattori, A., Arisaka, A., Hasegawa, S., & Saitoh, S. (2019). A novel splicing mutation in SLC9A6 in a boy with Christianson syndrome. Human genome variation, 6, 15.
29. Liu, X., Xie, L., Fang, Z., & Jiang, L. (2022). Case Report: Novel SLC9A6 Splicing Variant in a Chinese Boy With Christianson Syndrome With Electrical Status Epilepticus During Sleep. Frontiers in neurology, 12, 796283.
30. Zanni, G., Barresi, S., Cohen, R., Specchio, N., Basel-Vanagaite, L., Valente, E. M., Shuper, A., Vigevano, F., & Bertini, E. (2014). A novel mutation in the endosomal Na+/H+ exchanger NHE6 (SLC9A6) causes Christianson syndrome with electrical status epilepticus during slow-wave sleep (ESES). Epilepsy research, 108(4), 811–815.
31. Dong, Y., Lian, R., Jin, L., Zhao, S., Tao, W., Wang, L., Li, M., Jia, T., Chen, X., & Cao, S. (2023). Clinical and genetic analysis of Christianson syndrome caused by variant of SLC9A6: case report and literature review. Frontiers in neurology, 14, 1152696.
32. Zhang, H., Chen, X., Tan, H., Teng, Y., Liu, D., Wu, J., Duan, R., Liang, D., Li, Z., & Wu, L. (2023). The exploration of genetic aetiology and diagnostic strategy for 321 Chinese individuals with intellectual disability. Clinica chimica acta; international journal of clinical chemistry, 538, 94–103.
